# Outcome Evaluation of Burn Injury Management: A Study of Selective Traditional Home Remedies

**DOI:** 10.7759/cureus.45847

**Published:** 2023-09-24

**Authors:** Ziyad Alharbi, Eyas Farran, Jumana Akbar, Orjowan Abid, Zain Albukhari

**Affiliations:** 1 Plastic Surgery and Burn Unit, Dr. Soliman Fakeeh Hospital, Jeddah, SAU; 2 Clinical Sciences, Fakeeh College for Medical Sciences, Jeddah, SAU; 3 Medicine and Surgery, Fakeeh College for Medical Sciences, Jeddah, SAU; 4 Medicine, King Abdulaziz University Faculty of Medicine, Jeddah, SAU; 5 Medicine and Surgery, Batterjee Medical College, Jeddah, SAU

**Keywords:** scar evaluation, vss, cosmetic outcome, burn management, burn injuries, home remedies

## Abstract

Background

Clinicians classify burns as epidermal, partial thickness (superficial and deep), or full thickness, according to the depth of tissue damage. Although skin is considered the largest organ in the human body, studies investigating burns, their types, and their management has revealed that the background knowledge of burn aid the community possesses remains unsatisfactory. Thus, in this study, we aimed to evaluate the effect of various traditional home remedies, taking into account the type of burns and the nature of the remedies used from a cosmetic point of view.

Materials and methods

This is an original retrospective study conducted at Dr. Soliman Fakeeh Hospital in Jeddah from June through December 2022. Using the Vancouver Scar Scale (VSS), eligible patients who met our inclusion criteria were invited to participate in the study after a review of their patient history, an assessment of basic vital signs, and a physical examination.

Results

Fifty-two participants met our inclusion criteria and successfully completed the study. A total of 80 wounds of varying severity in various locations were evaluated. Participants were divided into three categories according to VSS scores indicating good, intermediate, or poor healing. None of the eight cases treated with water resulted in poor healing. However, tomato paste resulted in poor healing for six cases (60%) but moderate and good healing for two cases (20%).

Conclusion

The safest and most effective initial management for burns among all the reviewed remedies was the application of cool running water, followed by seeking medical attention for evaluation and proper treatment, whereas tomato paste had a markedly poor effect.

## Introduction

The skin is considered the largest organ in the human body; it serves as a protective barrier in addition to its many other vital functions for survival [1]. Burns are potentially harmful lesions that can occur at any time and have a variety of negative repercussions, including physical and occupational harm, loss of functionality, cosmetic deformity, and psychosocial harm [2]. The term "burn" is defined as an injury to the skin or other organic tissue that is primarily caused by thermal or other acute trauma. It occurs when one or more of the skin cells or other tissues are destroyed by hot liquids (scalds), hot solids (burns of contact), or flames (burns of fire) [3].

Describing burns as first, second, or third-degree alone does not adequately convey the significance of a burn injury. Due to the ambiguous and inconsistent interpretation of these words, they may be misleading [1]. Burns are also classified as epidermal, partial thickness (superficial and deep), or full thickness according to the depth of tissue damage. Fourth-degree burns extend beneath the subcutaneous tissues and involve fascia and/or muscle [4]. Because they cause an estimated 180,000 deaths each year, burns are a major public health concern worldwide. Almost two-thirds of these occurrences occur in the WHO regions of Africa and Southeast Asia, with the majority occurring in low- and middle-income countries [5]. The observed situation is made worse by the fact that Saudi Arabia has weak burn first-aid practices and a significant prevalence of traditional home remedies [6,7].

Saudi Arabian respondents to a study conducted in Majmaah reported having limited knowledge of first aid despite possessing bachelor's degrees and being generally well-educated [7]. Another study conducted in the Al-Baha region showed that the majority of participants (73.6%) had inadequate knowledge of first aid for burns, while only 26.4% had adequate knowledge [8]. In contrast, a 2011 survey of New South Wales residents conducted to assess their knowledge of burns and first aid revealed that only a minority were aware of correct burn first-aid procedures [9]. A study conducted in Kumasi utilizing 85 different substances, such as sand, muddy water, starch, corn dough, cow dung, egg white, calamine lotion, gentian violet, ointments, creams, and toothpaste, revealed diverse knowledge of first-aid techniques and administration [10].

Various limitations regarding first aid and initial care for burn patients have also been identified. Consequently, patients encounter numerous challenges during recovery, including more comorbidities, higher mortality rates, and longer hospital stays [11]. The early management of burn cases represents one of the biggest challenges and definitely reflects the degree of morbidity and mortality [10]. Cooling the burn surface is one of the most traditional forms of care and has been used for decades. However, new management strategies have been advised over time based on advanced assessment, burn site, and burn degree [12,13].

A study conducted in Vietnam revealed that only a minority of physicians had participated in emergency burn management training courses [11]. Another study carried out in a hospital in Lahore, Pakistan, showed that few of the parents who presented to the ER department with their children suffering from burn injuries had previous knowledge of how to manage burns [14]. In Northern Australia, a study showed that a limited number of total cases were less likely to perform burn first-aid interventions [9]. A 2017 study in Al-Madinah City, Saudi Arabia, revealed that parental knowledge of burn, injury, and fracture first aid is inadequate [15]. Another study conducted in 2018 in Riyadh, Saudi Arabia, concluded that the level of knowledge and awareness among parents regarding burn first aid was insufficient, finding that only 6% of 300 parents had an adequate awareness level of burn first aid. All the others, which represented the majority of the parents, relied on inappropriate myths to manage burns [16].

Studies about burns, their types, and methods of management have revealed the truth about the community's background knowledge of burn aid, exposing that the average level of knowledge is unsatisfactory and calls for improvement [17]. While the body of literature on first-aid knowledge and the management of particular populations is expanding, no published studies have sought to assess the effectiveness of appropriate burn management and its influence on outcomes. In this study, we evaluated initial patient management practices in Jeddah, Saudi Arabia, taking into account the type of burns and the nature of the remedies used.

## Materials and methods

Materials and methods

This is an original retrospective study conducted at one of the largest private hospitals in the Middle East, Dr. Soliman Fakeeh Hospital in Jeddah, on patients with different types and levels of burns who presented to outpatient clinics. An ethical approval request was submitted and approved by the institutional review board of Dr. Soliman Fakeeh Hospital (Approval No.:232/IRB/2021), and from June through December 2022, a total of 68 people were recruited. All participants gave their explicit written consent prior to the collection of data after being fully informed of the objectives of the study and assured that only the authors would have access to the information.

Inclusion/exclusion criteria and timeline

**Figure 1:**
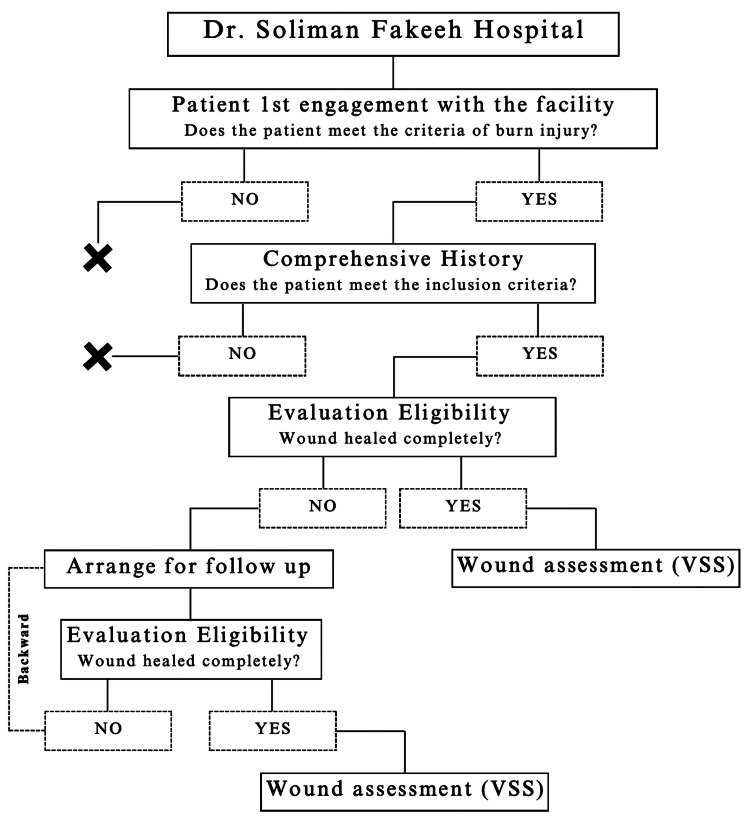
Timeline and criteria for conduction VSS - Vancouver Scar Scale

All patients who presented to the outpatient clinic were evaluated for eligibility for this study by the resident staff. Eligible patients were then invited to participate in the study. Patients with a burn incident of any type who presented to Dr. Soliman Fakeeh Hospital during the six-month period from June through December 2022 and who used any type of remedy as a first intervention before seeking medical advice were included in the study. We excluded patients with a history of chronic illness, such as autoimmune or systemic diseases, which might have affected the course of wound healing and the overall outcome. Their evaluation included a thorough review of their patient history, an assessment of basic vital signs, and a physical examination (Figure 1).

Assessment basis

The assessment was not centered on demographic characteristics but rather on study-specific variables, such as the degree and type of burn, the time of incidence, the time since the first home intervention, the nature of intervention, and the frequency of application, as well as the results for the Vancouver Scar Scale (VSS) shown in (Table 1). The scale considers four physical characteristics of scars: vascularity, height (thickness), pliability, and pigmentation. Thus, the lowest possible score is 0 (for normal skin), and the highest potential value is 13 due to a lack of data, since physicians do not typically document patients' management of their burns but only the management once the patient seeks hospital consultation. In this regard, the authors chose to create digitalized checklists (solely for organizational purposes) to evaluate the patients' knowledge and management at the time of occurrence and while the study was being conducted.

**Table 1:** The Vancouver Scar Scale (VSS)

Scar characteristic	Score
Vascularity	
Normal	0
Pink	1
Red	2
Purple	3
Pigmentation	
Normal	0
Hypopigmentation	1
Hyperpigmentation	2
Pliability	
Normal	0
Supple	1
Yielding	2
Firm	3
Ropes	4
Contracture	5
Height	
Flat	0
< 2 mm	1
2-5 mm	2
> 5 mm	3
Total score	13

Statistical analysis

Data were entered into Microsoft Excel (Microsoft, Redmond, Washington), coded, and reviewed for accuracy prior to being exported to SPSS version 25.0 (IBM Inc., Armonk, New York) for analysis. The frequency and percentages of the categorical variables were utilized for the descriptive analysis. The quantitative variables were described using frequencies and percentages. The Chi-squared test was utilized to examine the association between the variable of interest (VSS) and the other independent variables (initial intervention, type of burns, and degree of burns). The Pearson correlation coefficient was employed to examine the link between VSS and each of the following: frequency of application and duration of application using the same modality. Utilizing linear regression, the factors impacting burn healing were predicted. An interval of 95% confidence was utilized, and a p-value of less than .05 was deemed statistically significant.

## Results

A total of 68 participants were eligible for the study and included, but 16 patients were ultimately dropped for a variety of reasons, including failure to follow up, refusal to continue the study, and the use of a combination of creams or ointments in addition to the initial modality. This left 52 participants who met our inclusion criteria and successfully completed the study. Male patients made up 53.8% of the population, while female patients made up 46.2%; the mean age was 40.9 years (SD=17.1) (Table 2). 

**Table 2:** Baseline demographic characteristics (N=52)

Variable	N (%)
Gender	
Male	28 (53.8%)
Female	24 (46.2%)
Education	
Educated	29 (55.8%)
Not educated	23 (44.2%)
Variable	Mean ± SD (min-max)
Age	40.9 ± 17.1 (15-84)

A total of 80 wounds of varying severity and in various locations were evaluated. Wounds were divided into four distinct categories according to the degree of the burn: epidermal, superficial partial thickness, deep partial thickness, and full thickness. Patients with multiple wounds of varying severity were also grouped. Participants were divided into three categories according to VSS scores. In the first group, wound scores of 0-3 indicated good healing, scores of 4-8 indicated intermediate healing, and scores of 9-13 indicated poor healing. The majority of participants fell into the 2nd-degree category, with 25% having superficial partial thickness and 43.8% having deep partial thickness. Among the participants, thermal contact was the leading cause of burns. Ice had been applied to 8.8% of wounds, tomato paste to 12.5%, honey to 12.55%, olive oil to 7.5%, milk to 7.5%, yogurt to 10%, toothpaste to 12.5%, coffee grounds to 17.5%, and water to 11.3%. The majority of burns were either well or moderately healed (42.5% and 45%, respectively). Their total mean VSS score was 4.5, the mean frequency of using the initial substance was 2.8 times, and the average number of days prior to seeking medical help was 3.5 (Table 3).

**Table 3:** Medical characteristics of burns (N=80) VSS - Vancouver Scar Scale

Variable	N (%)
Burn degree	
Epidermal	3 (3.8%)
Superficial partial thickness	20 (25.0%)
Deep partial thickness	35 (43.8%)
Full thickness	22 (27.5%)
Burn type	
Thermal	76 (95.0%)
Chemical	4 (5.0%)
Remedy type	
Coffee ground	14 (17.5%)
Toothpaste	10 (12.5%)
Tomato paste	10 (12.5%)
Honey	10 (12.5%)
Water	9 (11.3%)
Yogurt	8 (10.0%)
Ice	7 (5.0%)
Milk	6 (7.5%)
Olive oil	6 (7.5%)
VSS categories	
Good healing	34 (42.5%)
Moderate healing	36 (45.0%)
Poor healing	10 (12.5%)
Variable	Mean ± SD (min-max)
VSS	4.5 ± 2.7 (0-10)
Frequency	2.8 ± 2.2 (1-9)
Duration	3.5 ± 3.8 (1-17)

The association between the remedies used for burn therapy and the healing categories (excellent, moderate, and poor) is discussed in (Table 4). The correlation is statistically significant (Chi=5.36, p=.021). None of the eight cases treated with water resulted in poor healing, whereas tomato paste resulted in poor healing for six cases (60%) and moderate and good healing for two cases (20%).

**Table 4:** Relationship between initial treatment materials and burn healing *Statistically significant; # Fishers' exact test

Initial treatment * TVSS categories	Good healing	Moderate healing	Poor healing	Total	Chi	p-value
Water	7 (77.8%)	2 (22.2%)	0 (0%)	9 (100%)		
Ice	5 (71.4%)	2 (28.6%)	0 (0%)	7 (100%)	5.36	0.021*#
Milk	4 (66.7%)	2 (33.3%)	0 (0%)	6 (100%)		
Toothpaste	6 (60%)	2 (20%)	2 (20%)	10 (100%)		
Yogurt	4 (50%)	4 (50%)	0 (0%)	8 (100%)		
Tomato paste	2 (20%)	2 (20%)	6 (60%)	10 (100%)		
Honey	2 (20%)	6 (60%)	2 (20%)	10 (100%)		
Coffee grounds	4 (28.6%)	10 (71.4%)	0 (0%)	14 (100%)		
Olive oil	0 (0%)	6 (100%)	0 (0%)	6 (100%)		

## Discussion

The efficacy of home remedies for treating multiple injuries, including burns, has been widely studied, but their definitive effect on the outcome "cosmetic wise" has never been determined, as burns are one of the most common injuries in the world and place a significant burden on healthcare systems. Our research was conducted in Saudi Arabia, where burn injuries are prevalent by nature. Some studies have suggested that socioeconomic status and cultural background are associated with the prevalence of burn injuries, which makes the location of our study of particular interest due to its understudied cultural and social environment [17,18].

Once the type of burn has been identified, immediate action must be taken. The efficacy of exposing a burn to cool running water has been repeatedly demonstrated as the most important initial step [3,19,20]. Following the administration of first aid, the definitive treatment for less severe burns consists of antibiotic coverage and analgesics, whereas more severe cases necessitate additional treatments, including possible skin grafts and substitutes. In numerous cultures, it is widely believed that honey, olive oil, tomato paste, toothpaste, and coffee grounds, among other substances, can aid in the treatment of burn wounds. In the most severe cases, the use of alternative medicines may cause infection, sepsis, or even death [10,21-23].

Numerous studies have been conducted on the efficacy of these common home remedies, and they are constantly being evaluated for their potential benefits. In this context, we cannot completely rule out the efficacy of some of these substances, as they have their own special proprieties. Olive oil, for example, has antifungal and antibiotic properties [17,24,25], while honey has some intriguing properties that may speed up the healing process, reduce scar formation, and lower the risk of infection [2,26].

Applying water as quickly as possible and at the proper temperature produced the best results in our study. It is a known fact that victims of a burn benefit from the prompt removal of the cause and cooling of the injured area. Reducing the elevated temperature of the burned tissue improves the physiological response. It also provides important palliative care. Using ice as the primary cooling agent, on the other hand, can aggravate an injury by restricting blood flow to the affected area (cold-induced vasoconstriction) [12]. Similar effects were observed with milk and yogurt, among other modalities. While honey, olive oil, coffee grounds, and toothpaste yielded moderately satisfactory results, tomato paste produced the least desirable results.

Study limitations

The evaluation of the data was based on the wounds that were present in the hospital at the time of the study, which provided a broad notion of the effects of only a few types of remedies applied by various patients. Another crucial issue that needs to be addressed is the fact that in this study, comprehensive observation of all cases was not conducted, which means that numerous non-modifiable and other modifiable elements, such as the application method and commitment from participants, may have had an impact on the outcome, not to mention that this study relied on the expertise of experts in VSS assessment; thus, there may be some variance in the results. Therefore, we recommend broadening the investigation to include other types of materials at other public and private institutions.

## Conclusions

Our findings indicate that the safest and most effective initial management technique for burns among all the remedies reviewed is the application of cool running water, followed by seeking medical attention for evaluation and proper treatment. The most markedly poor effect resulted from the application of tomato paste.

Our reliance on healthcare providers, hospital emergency rooms, and local urgent care units has detracted from our understanding of the importance of having a community that is educated about the first-aid management of burns and emergency home incidents. Of course, it is inadvisable to risk treatment with ineffective home remedies; however, cool running water, the simplest and most readily available modality, has been repeatedly demonstrated to be the most effective. Burn accidents are among the most common injuries in Saudi Arabia and the world, which is why we must take precautionary and preventative measures and educate the public about the significance of primary interventions that the victim or a bystander can carry out until professional medical assistance is available.
